# Accounting for clustering in automated variable selection using hospital data: a comparison of different LASSO approaches

**DOI:** 10.1186/s12874-023-02081-6

**Published:** 2023-11-25

**Authors:** Stella Bollmann, Andreas Groll, Michael M. Havranek

**Affiliations:** 1 Competence Center for Health Data Science, Faculty of Health Sciences and Medicine, University Lucerne, Frohburgstrasse 3, 6002 Lucerne, Switzerland; 2 Institute of Education, University Zurich, Kantonsschulstrasse 3, Zurich, 8001 Switzerland; 3 Department of Statistics, TU Dortmund University, Vogelpothsweg 87, 44227 Dortmund, Germany

**Keywords:** LASSO, Feature selection, Multilevel model, Hierarchical model, Hospital quality indicators, Risk-adjustment, Nested data

## Abstract

**Background:**

Automated feature selection methods such as the Least Absolute Shrinkage and Selection Operator (LASSO) have recently gained importance in the prediction of quality-related outcomes as well as the risk-adjustment of quality indicators in healthcare. The methods that have been used so far, however, do not account for the fact that patient data are typically nested within hospitals.

**Methods:**

Therefore, we aimed to demonstrate how to account for the multilevel structure of hospital data with LASSO and compare the results of this procedure with a LASSO variant that ignores the multilevel structure of the data. We used three different data sets (from acute myocardial infarcation, COPD, and stroke patients) with two dependent variables (one numeric and one binary), on which different LASSO variants with and without consideration of the nested data structure were applied. Using a 20-fold sub-sampling procedure, we tested the predictive performance of the different LASSO variants and examined differences in variable importance.

**Results:**

For the metric dependent variable *Duration Stay*, we found that inserting hospitals led to better predictions, whereas for the binary variable *Mortality*, all methods performed equally well. However, in some instances, the variable importances differed greatly between the methods.

**Conclusion:**

We showed that it is possible to take the multilevel structure of data into account in automated predictor selection and that this leads, at least partly, to better predictive performance. From the perspective of variable importance, including the multilevel structure is crucial to select predictors in an unbiased way under consideration of the structural differences between hospitals.

**Supplementary Information:**

The online version contains supplementary material available at 10.1186/s12874-023-02081-6.

## Background

Performance metrics such as *Duration Stay* or indicators of the quality of care such as *Mortality Rates* are widely used to assess healthcare providers’ performance. A broad branch of research has identified predictors that explain differences in such measures both at the patient as well as the provider level.

Recent improvements in data availability have increased the number of healthcare-related variables as possible predictors of these performance metrics (e.g., with regard to quality of care, see [43]). Consequently, the use of automated feature selection methods has also increased in healthcare research. Statistical tools like the *least absolute shrinkage and selection operator* (LASSO) and Bayesian additive regression trees (BART) have thus become more and more popular to facilitate variable selection.

Focusing more specifically on the quality of care of hospitals, there are at least two important areas of research in which the selection of relevant predictor variables may be used: (i) the prediction of quality-related outcomes and (ii) the risk-adjustment of quality indicators.

With regard to the task of prediction, automated variable selection is frequently used for prediction of hospital costs (see e.g., [3, 50]), but has also more recently started to be used for the prediction of quality-related outcomes. Studies using automated feature selection such as the LASSO have demonstrated its superiority in prediction accuracy [29].

Considering risk-adjustment, the variables to be used are traditionally selected based on theoretical considerations or expert opinions. However, this established approach has been criticized for its intensive resource and time demands [13], lack of objectivity and transparency, poor to modest accuracy, and insufficient generalizability [29]. Therefore, automated feature selection using machine learning methods has become increasingly popular [22, 34] and has been shown to achieve similar (and in terms of generalizability even better) results than theoretical selections for the risk-adjustment of quality indicators [13, 36].

All in all, there has been great progress in collecting large amounts of data to identify predictors for quality of care using automated variable selection methods both for prediction and risk-adjustment. However, all these studies face a methodological challenge that remains unresolved: the nested structure of the hospital data. We will now first show how multilevel models are already widely used as a means to account for the clustered structure of the data. Then, we will demonstrate why multilevel models should also be used for automated variable selection.

### Multilevel models accounting for clustering in hospital data

No matter whether one is aiming to predict quality-related outcomes at the patient level or measuring quality indicators at the hospital level, the data sets that are analysed are structured in a similar way: patients or hospitalizations are nested within hospitals, while some variables are measured at the hospital and some at the patient level. Typically, a great amount of variability of the dependent variables (DV) of interest, like quality of care [40], mortality [15, 48], and readmissions [14], is found at the hospital level. Nevertheless, significant information is lost when data is simply aggregated at the hospital level. Therefore, the method of choice for analysing such nested data sets are the so-called multilevel models or random effects models (see e.g., [8]).

When it comes to pure predictive performance, studies are inconclusive as to whether including random effects improves the prediction of quality measures. While some studies did not find significant improvements when including random effects (e.g., for predicting the mortality of corona artery bypass graft (CABG) surgery [28]), better performance of random effects models has, for example, been found when predicting return-visits [49] and risk-adjusted mortality for trauma patients [10].

Regardless of predictive performance, it has frequently been noted that ignoring the multilevel structure of hospital data may lead to false conclusions, the so-called “ecological fallacy” (see e.g., [19, 20]). This is why using random effects models has previously been advocated (see e.g., [17]). In addition, it has been empirically shown that estimated relationships indeed change dramatically depending on whether hospitals are included as random effects or not [2, 31]. In risk-adjustment, it is particularly important to disentangle hospital-associated effects from patient-related influences, as risk-adjustment models are used to adjust quality indicators for the influence of differing patient samples of hospitals. Consequently, multilevel based risk adjustment models have been found to perform better than standard methods [26]. Therefore, random effects models have increasingly been used not only to analyse associations between predictors and quality outcomes (e.g., [4, 35, 40, 42]), but also for the risk-adjustment of quality measures like mortality rates for COVID-19 patients [5], risk-adjusted mortality and morbidity rates [17], emergency readmissions [14], and septic shock mortality [48].

Further evidence for the need to consider the multilevel structure of hospital data in risk-adjustment is provided by the fact that both the Centers for Medicare and Medicaid Services (CMS) as well as the American College of Surgeons (ACS NSQIP) have adopted a multilevel-based approach to risk-adjust their readmission rates [32] and adverse outcomes after surgery [11], respectively.

Based on the above, we conclude that there is relatively great unanimity regarding the general importance of accounting for the multilevel structure of hospital data for two main reasons: the improvement of a) predictive performance, and even more importantly, b) unbiased variable selection and interpretation (see next section).

### Accounting for clustering in variable selection

As discussed, there is relatively large agreement in the literature that multilevel structures (i.e. clusterings of hospital data) must be considered in principle. Likewise, we could see that this is already done for analyses with manually selected predictors. However, in automated feature selection, clustering of hospital data is not yet sufficiently taken into account even though the consideration of the cluster structure is of great importance here. Neglecting the nested structure of data may alter estimated relationships (i.e. effect sizes) between the dependent and independent variables. A change in the effect sizes of the independent variables in turn may lead to differences in variable importance (in terms of the order of the variables by the absolute size of their coefficients). This possibly leads to a) false interpretations, b) a bias in variable selection, and thus c) a bias in the subsequent risk-adjustment, for which the selected variables are used.

To address this research gap, we investigate already existing methods to see how well they are able to perform exactly at this interface: Automatic variable selection for predicting quality of care variables in (nested) hospital data. Therefore, we chose a method that is particularly suitable for variable selection, as we will describe in more detail below: We investigated a variant of the variable selection technique LASSO that incorporates the estimation of random effects (i.e., a LASSO for general linear mixed models (GLMMs, [25]). Our goals were, on the one hand, to examine whether taking hospital clustering into account provides better predictive performance, and on the other hand, to see whether it leads to a different set of selected variables. In the following chapter, we will first explain the idea of the LASSO for predictor selection in general before describing an alternative LASSO variant that accounts for the clustering of the data.

### LASSO for predictor selection

Penalized regression, such as ridge regression [30], and LASSO [47], is a widely used method to overcome the problem of many predictor variables or a low ratio of number of observations to number of variables (including the $n<<p$ case) and/or a high collinearity of variables. Unlike in ridge, where coefficient estimates can never actually reach zero, LASSO has the desirable feature to also enable variable selection. Hence, in our paper, where we are mainly interested in variable selection, we focus on LASSO.

Besides enabling variable selection, the LASSO method can also be helpful in situations where substantial multicollinearity between predictors exists. If there is a group of predictors among which the pairwise correlations are very high, then the LASSO tends to select only one predictor from the group (but does not care about which specific one is selected). If the multicollinearity is pronounced, it is recommended to switch to a combination of ridge and LASSO, i.e. an elastic net [51] penalized version. Accomodating the suggestion of an anonymous referee, we inlcuded one specific variant of elastic net into our analyses as a comparison, namely the “nearly LASSO variant” that sets the elastic net parameter $\alpha =0.99999$. For implementation of these methods, we use the R package glmnet [21].

It is worth noting that penalization approaches typically involve at least one tuning parameter that needs to be optimized. For example, the LASSO penalty (see appendix) includes the penalty parameter $\lambda$, which controls the overall strength of the penalization.

In the case of clustered data, however, it would also be necessary to account for potential cluster-specific and unobserved heterogeneity during the variable selection process. This is of particular importance in the case of hospital data, where there can exist substantial heterogeneity among providers (see e.g., [15, 48]). However, one typically does not want to include all individual hospitals as (categorical) fixed-effect predictors into the model, because, on the one hand, this drastically increases the number of variables and may produce estimation problems, especially if the sample is small and/or the variables are highly correlated. On the other hand, one is typically not interested in the individual hospital (dummy) effect parameters but rather in identifying the underlying causes for differences between hospitals and generalizing to the population of all hospitals. Nevertheless, we believe that it would be important to include individual hospital-effects into LASSO models because this may:

- make predictions more accurate;
- lead to less biased estimates for the predictors;
- and conversely to less biased risk-adjustment models for quality indicators.

Hence, the incorporation of random intercepts for hospitals into the regularized regression model may be the best solution for the problem at hand. One specific LASSO implementation including random effects is available in the glmmLasso R package [24] within the GLMM framework.

Subsequently, we present our investigation of the inclusion of hospitals as random effects in several variable selection scenarios using the glmmLasso R package and the comparison with the traditional approach without considering the nested data structure using glmnet.

## Methods

### Data source

We used a national health administration data set provided by the Swiss Federal Statistical Office. It contains all inpatient cases treated in Swiss hospitals in 2019 with their diagnosis codes (ICD-10-GM, [16]), procedure codes (CHOP, [45]), diagnosis-related groups (SwissDRG, [46]), and other clinically relevant variables, such as the admission and discharge conditions as well as demographic information like age and sex. We focused on three different patient populations: (i) *COPD:* patients with a main diagnosis of chronic obstructive pulmonary disease (*n* = 12,404), (ii) *stroke:* patients with a main diagnosis of stroke (*n* = 23,276), and (iii) *heart attack:* patients with a main diagnosis of myocardial infarction (*n* = 19,242). All patients were older than 18 years of age, but aside from the focus on the selected main diagnoses and the age of the patients, no additional restrictions were applied. Since the data are provided in a standardized form, they do not contain any missing values.

For all data sets, we predicted the continuous DV *Duration Stay* that indicates how many days a patient was hospitalized. Additionally, we predicted the binary DV *Mortality* that indicates whether a patient died during the hospitalization. In doing so, we aimed to examine how well the different LASSO variants can deal with both continuous and binary data. Because of the greatly increased computational cost of some procedures when using the binary variable (*Mortality*), the largest data set (*stroke*) was reduced to a random sample of 40% of the hospitalizations within that patient population.

For our different investigated dependent variables, we obtained the following descriptive statistics: The overall percentage of mortality is 3.39% in the COPD data, 6.67% in the stroke data, and 4.33% in the heart attack data. The mean Duration Stay is 10.49 (sd = 8.50) days for COPD patients, 15.87 (sd = 22.12) days for stroke, and 6.43 (sd = 7.41) for heart attack patients.

For the DV *Duration Stay*, only observations with values larger than zero were included. Moreover, the following data preparation procedures were performed for all three data sets: to avoid extreme collinearities, the variable with a lower bivariate correlation in relation to the respective DV was excluded from each pair of predictors that had an (absolute) correlation above .95 (indicating a threshold, above which we started to experience computational problems in some settings).

In addition, all predictors were scaled to have the same variance prior to model estimation, which is necessary for the proper use of LASSO techniques to avoid selection bias in favour of variables with higher variance (see appendix for a list of all candidate predictor variables).

### Performance measures

We used a 20-fold sub-sampling procedure to evaluate the predictive performance of the different LASSO methods that will be described in the next section. Since some of our models also contained random effect estimates for each hospital, which can be used for the calculation of the deviance, we decided to include the requirement that each hospital from the test data was also represented in the training data with at least one observation. This restriction is the reason why our sub-sampling procedure is not a traditional 20-fold cross-validation. For each sub-sample run, the entire data set was randomly split into two parts, so that 1/20 of the cases are chosen as test data and the rest as training data. This was repeated 20 times. However, the resulting test data are not necessarily mutually exclusive from one sub-sample run to the next. The resulting 20 training and test data sets were created once at the beginning for each of the three data sets and were then used for all analyses for greater comparability. Furthermore, whenever a variable had zero variance in one of the sub-samples, the variable was removed to make the scaling of all variables possible.

The DV *Duration Stay* was highly positively skewed and therefore needed to be log-transformed before the analyses. In the appendix, figures are provided showing the qq-plots of the original and log-transformed DV, respectively.

To assess predictive performances, for the DV *Duration Stay*, we used the root mean squared error (RMSE) on the test data, averaged over the 20 sub-samples:

1 $$\begin{aligned} RMSE = \sqrt{\frac{1}{n} \sum \limits _{i=1}^{n}{(y_i - \hat{\mu}_i})^2 } \end{aligned}$$

For the binary DV *Mortality*, we used four different measures of predictive performance:

(i) the area under the Receiver Operator Characteristic (ROC) curve (AUC, [27]). The ROC curve plots the True Positive Rate (TPR) against the False Positive Rate (FPR). The TPR is defined as

2 $$\begin{aligned} TPR = \frac{TP}{TP + FN} \end{aligned},$$

where TP is the number of cases that were correctly classified as positive and FN is the number of cases that were falsely classified as negative. And the FPR is defined as

3 $$\begin{aligned} FPR = \frac{FP}{TN + FP} \end{aligned},$$

with FP being the number of cases falsely classified as positive and TN being the number of cases correctly classified as negative.

(ii) the area under the Precision-Recall (PR) curve (AUPRC, [12, 23]). The idea of the PR curve is similar to that of the ROC curve, but it plots the recall on the x-axis and the precision on the y-axis. Recall is the same as TPR, whereas precision is what is also called the Positive Predictive Value (PPV):

4 $$\begin{aligned} PPV = \frac{TP}{TP + FP} \end{aligned}$$

The AUPRC was shown to be more informative than the AUC for imbalanced data (e.g. [41]).

(iii) the Brier score (BS, [7]), defined as the average square difference between observed values ($y_i$) and predicted probabilites ($\hat{p}_i$) (therefore equivalent to the mean squared error):

5 $$\begin{aligned} BS = \frac{1}{n} \sum \limits _{i=1}^{n}{(y_i - \hat{p}_i})^2 \end{aligned}$$

and (iv) the predictive Bernoulli likelihood (BL):

6 $$\begin{aligned} BL = \frac{1}{n} \sum \limits _{i=1}^{n} \hat{p}_i^{y_i}(1-\hat{p}_i)^{1-y_i} \end{aligned}$$

All those measures were averaged over the 20 sub-samples. Since the AUC is the most commonly used measure in medical sciences, we focused our interpretation on this and only reported the others as an addition. In contrast to the RMSE, the AUC is readily comparable across studies, with high values indicating good performance.^1^ Generally accepted guidelines for AUC values indicate that values above .70 can be considered as acceptable discrimination, and values above .80 as excellent discrimination (see e.g. [33]). However, those cut-off values should be interpreted with caution.

Additionally, for the DV *Duration Stay*, we examined how variable importance and thereby variable selection changes when hospitals are included. More precisely, we were interested in which variables are considered the most important for each LASSO variant. To this end, we registered the five variables from all 20 data sets with the largest absolute coefficients in the model selected via cross-validation and included information on how often each of them was among the five most important over the course of the 20 sub-samples. Hence, in line with the concept of stability selection [38], we define variable importance as the percentage a variable was selected in this process across the 20 sub-samples.

### Investigated LASSO variants

As previously mentioned, we generally distinguished between standard, fixed-effects-only LASSO models (via glmnet) and the LASSO variant including random effects (via glmmLasso). In addition, we included glmnet with fixed-effects for the hospitals as an intermediate solution for comparison with the two other approaches. Adaptive LASSO versions were also investigated in glmnet, but they lead to similar results and, hence, these are not presented here.

Consequently, the different models we compared were: (i) *No hosps*, in which a traditional LASSO is used without any hospital effects; (ii) *Hosps fixed*, in which the hospitals are included as a categorical predictor in a traditional fixed-effects LASSO model; and (iii) *Hosps random*, in which hospitals are included as random effects.

As mentioned above, the LASSO penalty includes the tuning parameter $\lambda$ that determines the strength of the penalization. Typical procedures for the tuning of $\lambda$ are *K*-fold cross-validation (CV), which is the default in the function cv.glmnet($\cdot$) from the glmnet package. Another alternative would be information criteria-based approaches using Akaike’s information criterion (AIC) [1] or the Bayesian information criterion (BIC) [44], also known as Schwarz’s information criterion, with the difference that BIC tends to favor sparser models.

Finally, in addition to the standard LASSO-estimates, we also calculated the so-called *post-LASSO* estimates, which are based on the idea of the relaxed LASSO [37] and were recently implemented in glmnet via the relax argument. These are also available in glmmLasso by setting the final.re=TRUE argument. The main idea of the post-LASSO is that once the variables are selected given a certain $\lambda$ from the investigated grid of penalty parameters, this set of “active” variables is used to re-fit an unregularized model (e.g., via conventional maximum likelihood). The optimal penalty parameter is then tuned based on these post-LASSO estimates. In contrast to the standard LASSO that we call *classic* in the following, this approach will be referred to as *post*. For all methods and data sets, we examined both estimation with the post-LASSO (*post*) and without (*classic*).

#### LASSO with only fixed effects

For both the *No hosps* and *Hosps fixed* models, we used the R implementation glmnet [21] to estimate a classical LASSO-penalized regression model. The glmnet algorithm internally selects the optimal $\lambda$ using the deviance (in case of a normally distributed DV, deviance corresponds to the MSE) in a 10-fold cross validation.

A suitable grid of $\lambda$ values on which this validation is performed is automatically chosen by the function. After the optimal $\lambda$ is selected, the LASSO is fitted again on the entire data. Please note that two options for model selection are provided: $\lambda _{min}$ is the $\lambda$ that yields the minimal deviance and $\lambda _{1se}$ is the largest value of $\lambda$ such that the error is within one standard error of the minimum. In addition, we also used AIC and BIC (as they are used in glmmmLasso, see below) as selection criteria and compared their results with $\lambda _{min}$ and $\lambda _{1se}$.

#### The LASSO with hospitals as random effects

For *Hosps random*, we use the R package glmmLasso [25]. Unfortunately, this package does not yet provide an automated mechanism for the specification of a suitable $\lambda$ grid on which to perform the internal cross validation. Therefore, we manually determined a $\lambda$ grid consisting of 100 $\lambda$ values, so that for the highest $\lambda$ in that grid not a single variable is selected and all corresponding coefficients are shrunk to zero. Within the first five $\lambda$ values of that grid, the number of selected variables increases to at least one. In each iteration of the algorithm, the fit from the previous iteration is handed over as a starting point for the fixed effects and random intercepts.

**Fig. 1:**
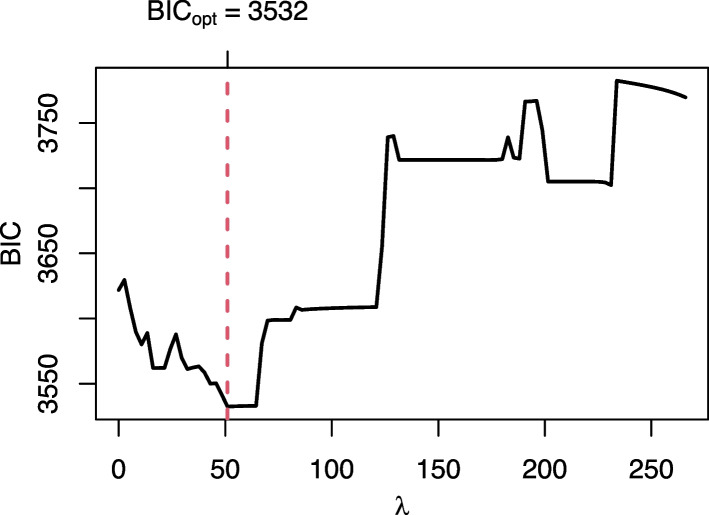
BIC plot for glmmLasso for one subsampled training fold of the COPD data

**Fig. 2:**
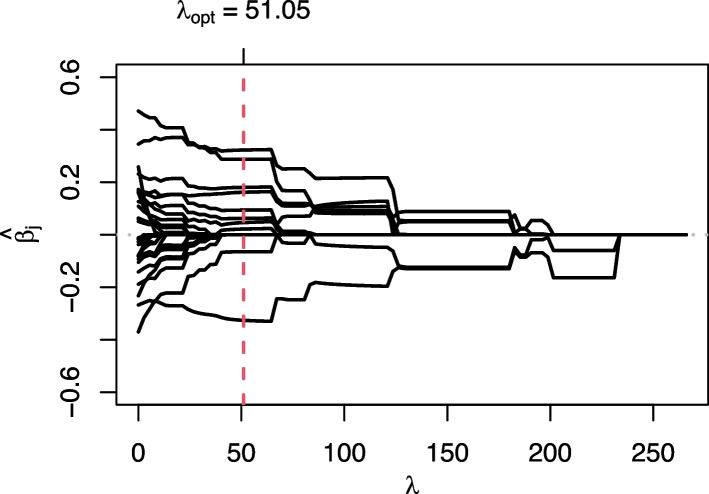
Coefficient paths plot for glmmLasso for one subsampled training fold of the COPD data

When using AIC or BIC as an optimality criterion in the tuning procedure, the model is estimated on the entire data for each $\lambda$ and the resulting AIC or BIC are obtained. Predictions from the model with the lowest AIC or BIC, respectively, are then used to calculate the predictive performance. For illustration purpose, we show the BIC plot for the determination of the optimal $\lambda$ as well as the corresponding coefficient path plot (see Figs. 1 and 2) for one arbitrary training data set (using COPD patients). In the BIC plot, the resulting BIC is shown for each $\lambda$ from the $\lambda$ grid (in this example from 266 to 0). The lowest BIC obtained is for a $\lambda =51.05$. In Fig. 2, the paths of the estimated coefficients for all variables are shown for each $\lambda$. It can be seen that the worst model fit is that for $\lambda$ values close to the maximum, where all coefficients are shrunk to zero and, hence, no variable is selected. Reading from right to left, the BIC then decreases and reaches its minimum for $\lambda \in [50; 60]$ before increasing again.

When using CV-based tuning, the training data is split into 5 folds to be used in a 5-fold cross-validation. The optimal $\lambda$ is determined based on the deviance. Then, the model is estimated again on the entire training data using this optimal $\lambda$ and predictions are obtained from that final model on the test data to calculate the predictive performance. In line with the models *No hosps* and *Hosps fixed*, the model resulting from this optimal $\lambda$ will be called $\lambda _{min}$. Unfortunately, in the glmmLasso package, the $\lambda _{1se}$ option is not available and will therefore not be reported. All analyses were performed using the statistical software program R [39].

## Results

### Predictive performance

#### Duration stay

**Fig. 3:**
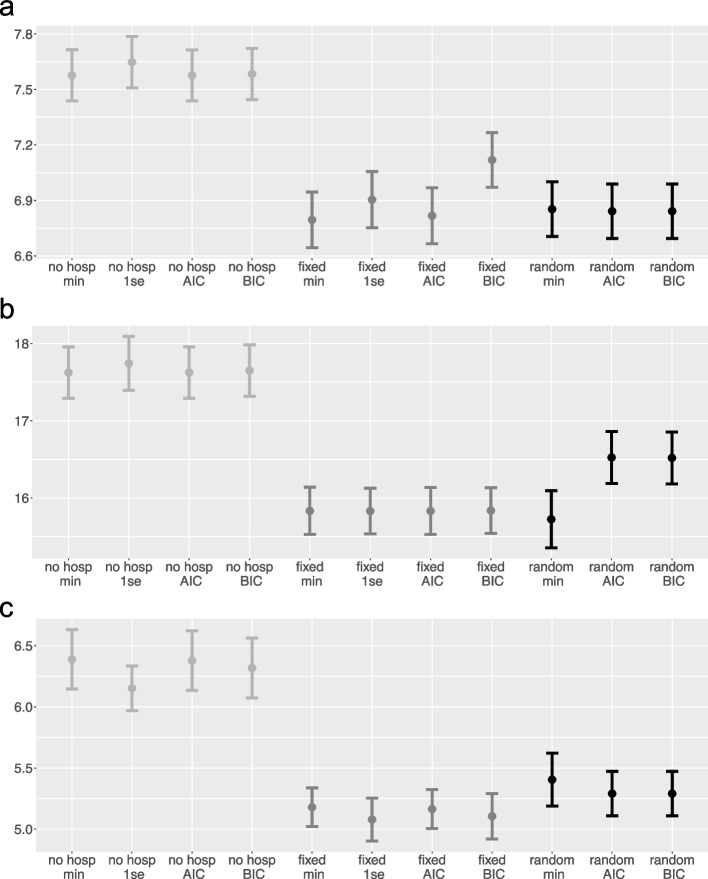
RMSE (means and standard deviation bars) for the DV *Duration Stay* in the three data sets, respectively

The MSEs for all classic variants (in contrast to the post-LASSO variants that we will briefly discuss in the next paragraph) are summarized in Fig. 3 in three subfigures for the three data sets, respectively. The results are based on averaged MSEs over the 20 sub-sample iterations. In the COPD data, it can be seen from Fig. 3a that including the hospitals, whether as fixed or as random effects, leads to considerably better results. However, there is little difference between the different variants of including the hospitals and the various optimization criteria within those variants, except that the MSE is larger for the *Hosps fixed BIC*. Taking a closer look at the different conditions, we can see that within glmnet, the $\lambda _{1se}$ optimization criterion seems to achieve better results than $\lambda _{min}$ in both variants. In the stroke data, including hospital effects also revealed better predictive performance than not including them (see Fig. 3b), and within the *hosps random* variant, AIC and BIC performed worse than CV. Figure 3c shows that in the heart attack data, *Hosps fixed* performed best on average and the variants without the inclusion of hospital effects performed the worst. *Hosps fixed* is slightly better than *Hosps random* in this data set, but the effect is negligible.

The post-LASSO variant on average performed worse than the classic variant for both DVs, with higher MSEs or lower AUC values, respectively, and larger standard errors. For this reason, we do not go into detail regarding their interpretation. However, an exemplary plot is shown in the appendix.

The analyses for the “nearly LASSO variant” suggested by an anonymous reviewer resulted in exactly the same predictive performance values as the LASSO variant for all data sets and all outcome variables. We therefore refrain from reporting them here.

#### Mortality

**Fig. 4:**
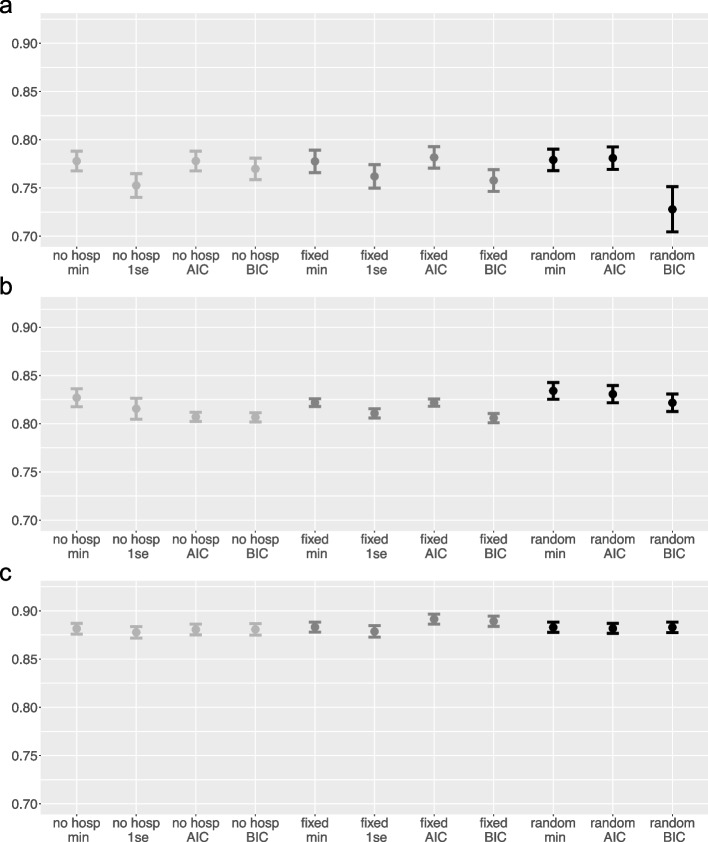
AUC (means and standard deviation bars) for the DV *Mortality* in the three data sets, respectively

Figure 4 shows mean AUC values and standard error bars for the results of the different LASSO variants on the binary DV *Mortality* in the three data sets. According to generally accepted thresholds (see e.g., [33]), all variants achieved excellent performance in the stroke data and even better performance in the heart attack data. In the COPD data, the performances were slightly lower and showed more variability. Additionally, the AUC values showed only small differences between the different variants. In the stroke data, a slight advantage of the *Hosps random* model can be observed. Most of the differences seem to occur when comparing different optimization criteria, with $\lambda _{min}$ and *AIC* criteria performing a little better than the others, at least in the first two data sets. This finding could be because those criteria in general select more variables than the other two, which appears to be the better solution in those data sets. In the stroke data, for example, *No hosps* with $\lambda _{min}$ variant selects 39.9 variables on average, with $\lambda _{1se}$ 17.6, with *AIC* 45.2, and with *BIC* 35.95. *Hosps random* with $\lambda _{min}$ (which achieves the best performance) selects 44.25 variables on average, with *AIC* 42.7, and with *BIC* 18.8 variables on average.

The results are less clear when comparing the Brier score or the predictive Bernoulli likelihood between the variants. Here, almost no systematic variation between mean performance measures could be found (see Table 1). However, the highest performance per data set and criterion (printed in bold in Table 1) was always found in a model which accounts for the hospital effects in some way.

**Table 1:** Averaged performance measures over 20 folds for the DV *Mortality*

	No hosps				Hosps fixed				Hosps random		
	$\lambda _{min}$	$\lambda _{1se}$	*AIC*	*BIC*	$\lambda _{min}$	$\lambda _{1se}$	*AIC*	*BIC*	$\lambda _{min}$	*AIC*	*BIC*
**COPD data**											
**AUPRC**	.12 (.009)	.11 (.008)	.12 (.009)	.11 (.008)	**.15** (.017)	.12 (.013)	.14 (.012)	.12 (.009)	.14 (.020)	.12 (.011)	.12 (.011)
**Brier**	.032 (.001)	.032 (.001)	.032 (.001)	.032 (.001)	.032 (.001)	.032(.001)	**.031**(.001)	.032 (.001)	.032 (.001)	.032 (.001)	.032 (.001)
**PBL**	.938 (.001)	.937 (.001)	.938 (.001)	.937 (.001)	**.939** (.001)	.938 (.001)	.938 (.001)	.936 (.001)	.938 (.002)	.938 (.002)	.935 (.002)
**Stroke data**											
**AUPRC**	.28 (.021)	.27 (.020)	.28 (.020)	.28 (.020)	.25 (.020)	.28 (.021)	.18 (.026)	.21 (.030)	**.29** (.024)	.28 (.026)	.28 (.026)
**Brier**	.055 (.002)	.056 (.001)	.055 (.001)	.055 (.001)	.055 (.001)	.055 (.001)	.055 (.001)	.055 (.001)	**.052** (.002)	.055 (.002)	.055 (.002)
**PBL**	.892 (.002)	.890 (.002)	.892 (.001)	.890 (.001)	.893 (.001)	.892 (.001)	.893 (.001)	.889 (.001)	**.901** (.004)	.893 (.004)	.889 (.003)
**Heart attack data**											
**AUPRC**	.28 (.010)	.28 (.010)	.28 (.010)	**.29** (.011)	**.29** (.011)	.28 (.010)	.31 (.013)	.28 (.011)	**.29** (.010)	**.29** (.010)	**.29** (.010)
**Brier**	.034 (.001)	.035 (.001)	.034 (.001)	.034 (.001)	.034 (.001)	.035 (.001)	**.033** (.000)	**.033** (.001)	.034 (.001)	.034 (.001)	.034 (.001)
**PBL**	.931 (.001)	.930 (.001)	.932 (.001)	.930 (.001)	.932 (.001)	.930 (.001)	**.933** (.001)	.930 (.001)	.932 (.001)	**.933** (.001)	.931 (.001)

*Note.* *AUPRC:* Area under the Precision-Recall curve; *Brier:* Brier score; *PBL:* predictive Bernoulli likelihood. In brackets: Standard errors. The highest number per line is marked in bold

### Variable importance

A summary of the variable importance (defined as the rate of being among the five most important variables in the model selected by CV across all sub-samples) for the COPD data is provided in Table 2. The variable importances for the other two data sets are shown in the appendix.

**Table 2:** Variable importances for the CV selected model concerning COPD data for the DV *Duration Stay*

Variable	no hosps	hosps fixed	hosps random
*Adm from hospital*	100% (.16)	100% (.11)	100% (.10)
*Pre-MDC*	100% (.22)	100% (.17)	100% (.19)
*Part medical*	100% (-.21)	100% (-.19)	100% (-.23)
*MDRG E65*	100% (.25)	100% (.13)	100% (.18)
*elix 23*	0% (–)	100% (.11)	100% (.11)
*Planned admission*	100% (.13)	0% (–)	0% (–)
*elix 1*	0% (–)	100% (.07)	0% (–)
*elix 30*	0% (–)	100% (.07)	0% (–)
*elix 24*	0% (–)	70% (.06)	0% (–)
*Emergency*	0% (–)	30% (-.06)	0% (–)

*Note.* Rates of being among the five most important variables (according to absolute values of regression coefficients) across the 20 sub-samples in percent; in brackets: mean estimated regression coefficient over all sub-samples

The results show that the variable importances differ between the different variants depending on the inclusion of hospital effects. Particularly interesting is the variable *Planned admission*. In both the COPD and heart attack datasets, this variable has an importance of 100% in the *No hosps* variant that drops to 0% in the two variants where hospitals are included. This variable specifies whether patient admissions were planned and thus distinguishes elective from emergency admissions. It has a large positive effect on *Duration Stay* whenever hospitals are not specifically included in the variable selection. This may be because the frequency of planned admissions can be a structural difference between hospitals and, therefore, the importance of this variable disappears in models where hospital effects are considered as well. In the COPD data for example, 14.49% of hospitals have no planned admissions at all, while 22.46% of hospitals have only planned admissions. This makes sense with regard to the healthcare system in Switzerland, where certain hospitals (such as private clinics) can focus on planned (i.e., elective) patients (compared to emergency patients), while other hospitals (such as general hospitals) cannot do so because of their service mandate. Thus, it could be argued that not including hospital effects in the feature selection leads to a biased selection of variables, such as *Planned admission*, which implicitly distinguish between the service mandates of hospitals (rather than the selection of more suitable variables directly linked to the patient characteristics). In the stroke data, *Planned admission* similarly becomes less important when including hospital effects, but only when including them as random effects.

A contrary effect can be observed for the variable *elix23* that denotes the Elixhauser comorbidity group “weight loss” according to the categorization by Elixhauser [18], which has a positive effect on *Duration Stay*. In all three data sets, this variable becomes far more important when hospitals are included, which may suggest that it is not as much associated with *Duration Stay* on a between-hospital level as it is on a within-hospital level. Such effects can be caused by very different classification thresholds in different hospitals. Or it could be that the importance of this (and similar) variable(s) related to patient characteristics become more relevant and are thus selected more frequently if other variables associated with the service mandate of hospitals (such as the variable *Planned admission*) are selected less often.

In the stroke data, there are a couple of other variables that are less important when including hospitals into the model in general. Those are: *Emergency*, indicating whether a patient was admitted as an emergency, *From home*, indicating whether a patient was admitted from home, and, to a lesser extent, *From hospital*, indicating whether the patient was admitted from another hospital. One can also see that the Elixhauser groups are only important when hospital effects are taken into account, similar to what has been found in terms of the comorbidity group “weight loss”. Rather, it seems that either hospitals differ a lot in whether their patients have such comorbidities or they differ in how conscientiously these comorbidities are coded in their administrative data. Or alternatively, as pointed out above, it could be that such variables related to patient conditions become more important, when variables associated with the service mandate of hospitals are selected less frequently (due to the inclusion of hospital effects). The specific interpretation of the individual effects of the selected variables is complicated by the fact that they would have to be interpreted depending on the effects of all the other variables. Thus, we refrain from going into detail in our interpretation of the variables, but the main finding across all investigated patient populations is the fact that different variables are selected depending on whether the hospital effects are considered or not.

**Fig. 5:**
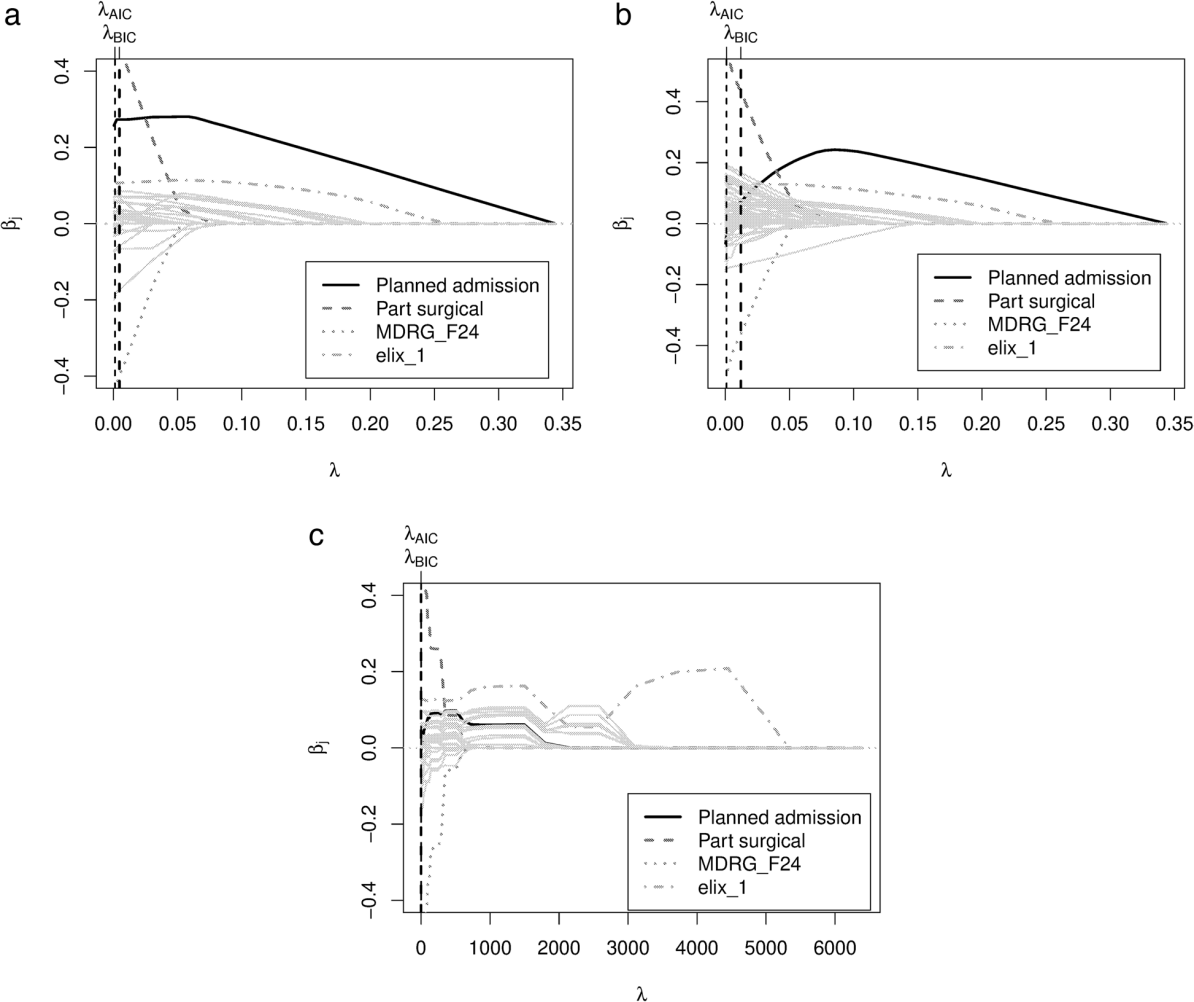
Coefficient paths plot for heart attack data DV *Duration Stay*

To further illustrate the trajectories of a few exemplary variable importances, we also created three coefficient path plots for a randomly selected sub-sample of the heart attack data set (see Fig. 5). As in Fig. 2, these plots show how the magnitude of the coefficients changes with decreasing penalization (lambda) (read from right to left). In Fig. 5, four of the variables are specially highlighted. Thus, one can clearly see how the variable *Planned admission* rises very quickly in the *No hosps* variant (Fig. 5a) and remains at a high level, while in the *Hosps fixed* variant (Fig. 5b), it also rises sharply to begin with but then falls again with an increasing number of hospitals inserted. In the *Hosps random* condition (Fig. 5c), where hospitals are included from the start, this variable is less important from the beginning. The other three highlighted variables, on the other hand, show very similar patterns in all three variants.

## Discussion

The purpose of this study was to account for hospital clustering while performing automated variable selection with a particular regard to the predictors of hospital performance and quality of care. We applied three different LASSO variable selection variants: i) without hospitals, ii) with hospitals as fixed effects, and iii) with hospitals as random effects on three different patient data sets. We first examined how their predictive performance differs and second how their variable selection differs.

We found an improvement in predictive performance in models that include hospitals compared to those that do not include them in most scenarios. This is in line with some previous findings (e.g., [10, 49]), but contradicts others (e.g., [28]). Therefore, it may be concluded that whether accounting for hospital clustering improves predictive performance depends on the data, but it at least does not distort predictive performance.

More importantly, however, we found that variable importance changes when hospitals are included. Some variables go from being among the top five for all 20 sub-samples to no longer being in the top five for any of the sub-samples when hospital effects are included. These findings are in line with results from other studies that examined predictive performance after the manual selection of features (e.g., [2, 31]). In these previous studies, it could also be shown that the estimates of the association between variables differed considerably, depending on whether or not random effects were included. Such differences can, of course, lead to completely different interpretations of dependencies between predictor variables and the DV, as has already been stressed previously [19, 20]. Another consequence of our results is that the comparison of selection results from different approaches (i.e., with or without the consideration of hospital effects) could be used to learn about dependencies within the data in a data-driven way. More specifically, it may support researchers to assess which variables are mainly associated with hospital differences and which are primarily related to patient differences.

Based on these findings, we recommend that the natural clustering of hospital data already should be considered when selecting predictor variables for prediction purposes or the risk-adjustment of quality indicators, in contrast to only considering it in the final modeling stage (once the predictors have been selected). This approach leads to predictions that are better or at least as good as when not considering hospital effects. In addition, when hospital effects are not included, interpretations of the importance of predictor effects and therefore dependencies among variables can be distorted. This may be of particular importance for the selection of variables for the risk-adjustment of quality indicators, as risk-adjustment aims to control for patient characteristics but not treatment differences among hospitals. Furthermore, as has been shown in previous studies, the performance of risk-adjustment methods increases when including random effects [26]. As mentioned earlier, variable selection for risk-adjustment procedures is increasingly conducted in a data-driven way, especially since the traditionally used theory- or expert-based approaches have come under criticism (e.g., [13, 29, 34]). However, in our opinion, doing so correctly would entail making use of the existing possibilities (e.g., the R package glmmLasso) to include the naturally occurring hierarchical structure of the data. This may be especially important if, as is commonly the case, hierarchical modeling is intended to be used for risk-adjustment after the feature selection has been conducted.

For the different optimization criteria (AIC, BIC, CV), no major difference was found. The AIC/BIC based approaches have the advantage of being significantly faster because of their lower computational costs. However, in cases where the computation speed is not important, it may be advisable to use the default setting in glmnet, which is cross validation, and to use this for comparisons with glmmLasso as well.

It has to be stressed that the focus of this paper was on variable selection and interpretation in important healthcare application settings. As such, we target researchers who are interested in detecting predictors and interpreting their association with the dependent variable. An important use case is the variable selection for risk-adjustment models of quality indicators. Linear models are typically used here because interpretation and understandability are essential. Including hospitals as fixed effects would not be suitable in this situation, because the developed models are designed to describe relationships between patient characteristics and the outcome variable independent of the hospitals’ performance. For this reason, our *Hosps fixed* models were merely used as comparison in this study, but our main goal was to contrast the *No hosps* and *Hosps random* models. However, if one is mainly interested in predictions of the dependent variable on new data, there are other machine learning approaches that are worth considering as e.g. XGBoost [9] or Random Forest [6], which could include the hospitals as simple fixed effects.

In addition, it is worth noting that the above-mentioned tuning parameter alpha for elastic net does not have to be set to 0.99999 but can also be tuned in a data-driven way. This tuning process can be programmed using the cva.glmnet function in the glmnet add-on package glmnetUtils. However, one should be aware that especially with large data sets, the additional tuning of a further parameter means additional computational costs. An even more important reason why we did not include this variant in the present work is that for glmmLasso no elastic net version is yet implemented and, hence, it can only be investigated for the glmnet versions (i.e. the approaches without random effects). For this reason, the classical elastic net version cannot be compared for the different modeling strategies of the hospital effects, which were investigated in this study.

### Study limitations

It must be noted that the performance of glmmLasso still depends greatly on the quality of the $\lambda$ grid. Another related technical limitation is the fact that the approach used in the implementation glmmLasso is computationally expensive and thus time-consuming.

Moreover, there is a general limit to how much a variable selection process can be automated. Even when using a technique that can deal with a great number of variables like the LASSO, the candidate predictors must be chosen in some way. Thus, a domain expert needs to make certain decisions regarding the variables, such as which data sources to use; how to operationalize, calculate or transform the variables; and which variables to exclude. Theoretical or domain-specific knowledge will always be required for this purpose. Similarly, when interpreting the results, an expert is needed to provide insights into the underlying causes of the findings and/or the possible dependencies among the variables.

## Conclusion

Our research presents an approach to include the clustering of hospital data in variable selection for predicting quality-related outcomes and risk-adjusting quality indicators. The predictive performance of the investigated glmmLasso (under the consideration of hospital effects) is at least as good or better than that of the more traditionally used glmnet (without the inclusion of hospital effects). Furthermore, in line with previous studies, we have found that neglecting the clustered structure of the data may lead to biased parameter estimates and potentially false conclusions. This is especially important in the context of risk-adjustment, where a careful selection of variables is pivotal. Therefore, we conclude that automated feature selection, which considers the nested data structure, is the method of choice for the prediction of quality-related outcomes and the risk-adjustment of quality indicators.

### Supplementary Information

**Additional file 1.**

## Data Availability

The data that support the findings of this study are available from the Swiss Federal Office of Statistics (contact via gesundheit@bfs.admin.ch) but restrictions apply to the availability of these data, which were used under license for the current study, and so are not publicly available. Data are however available from the corresponding author (stella.bollmann@ife.uzh.ch) upon reasonable request and with permission of the Swiss Federal Office of Statistics. The R code that was used for the analyses of this paper is publicly available (https://osf.io/3wbk7/?view_only=b58535f1952b43ef886dc7b488950b2a).

## Footnotes

1 Note that it can be shown that the sample variance and standard deviation are, respectively, the MSE and the RMSE of the naive predictor. Therefore, scaled MSE and RMSE represent the relative efficiency of the current predictor compared to the naive one, which, in principle, makes them comparable across different studies provided that the unit of measurement is the same.
