# Supplementary material for: Accounting for clustering in automated variable selection using hospital data: a comparison of different LASSO approaches
Source: BMC Med Res Methodol. 2023 Nov 25;23:280. doi: 10.1186/s12874-023-02081-6 (PMC10675967; doi:10.1186/s12874-023-02081-6)
Supplement: Supplementary file 1 — Additional file 1. [file 12874_2023_2081_MOESM1_ESM.zip › Appendix_1.pdf]

0.1 The LASSO

In situations of many predictor variables or a low ratio of number of observations to number of variables (including the $n << p$ case) and/or a high collinearity of variables, standard regression approaches typically become problematic and unstable, or even infeasible. One solution for this problem is the use of regularization techniques, in particular penalized regression, such as ridge regression [31] and LASSO [48]. The idea of the penalization is to add a penalty term to the actual estimator of the regression coefficients. This ensures that when minimizing the residual sum of squares, a penalty is imposed on the size of the coefficients. The penalization term that is added to the residual sum of squares in ridge regression is:

$$J_{ridge}(\boldsymbol{\beta}) = \lambda \sum_{j=1}^{p} \beta_j^2 \,, \tag{1}$$

Thus, the squared regression coefficients are penalized and, hence, shrunk towards zero.

However, in ridge regression the coefficient estimates can never actually reach zero. So, in cases where variable selection is desirable, a different penalty term needs to be considered. Here, the LASSO has turned out to be very useful. Instead of penalizing the squared regression coefficients, it addresses their absolute values:

$$J_{lasso}(\boldsymbol{\beta}) = \lambda \sum_{j=1}^{p} |\beta_j| \,. \tag{2}$$

Though this penalty term comes with the desirable feature of enabling variable selection, as the coefficient estimates can be shrunk towards zero due to the absolute value function, the corresponding optimization becomes more cumbersome and the LASSO estimator does not exist in closed form. Instead, numerical methods must be used. Here, we use the R package `glmnet` [21].
