# Supplementary material for: Accounting for clustering in automated variable selection using hospital data: a comparison of different LASSO approaches
Source: BMC Med Res Methodol. 2023 Nov 25;23:280. doi: 10.1186/s12874-023-02081-6 (PMC10675967; doi:10.1186/s12874-023-02081-6)
Supplement: Supplementary file 1 — Additional file 1. [file 12874_2023_2081_MOESM1_ESM.zip › Appendix_Table1.pdf]

Table A.1 Variables for all three data sets.

Type	Variable name	Description	Data set(s)
Demographics	Nationality		COPD, HA, S
	Gender		COPD, HA, S
	Age		COPD, HA, S
Admission from	From home		COPD, HA, S
	From home w SPITEX		S
	From different facility	From care home or nursing home	COPD, HA, S
	From hospital	Different / same hospital	COPD, HA, S
	From rehab	Rehab clinic	S
Admission type	Emergency		COPD, HA, S
	Planned admission	Registered / planned admission	COPD, HA, S
	Internal transfer		S
	Transfer 24h	Transfer within 24 h	HA, S
Admission authority	Self adm		COPD, HA, S
	Ambulance	Ambulance	COPD, HA, S
	Doctor	Doctor	COPD, HA, S
Partition of case	Part surgical		COPD, HA, S
	Part medical		COPD, HA, S
	Part other		HA
MDC	Pre-MDC	All diagnoses/procedures that are related to transplants	COPD, HA, S
	MDC1	Diseases and Disorders of the Nervous System	S
	MDC4	Diseases and Disorders of the Respiratory System	COPD
	MDC 5	Diseases and Disorders of the Circulatory System	HA
MDRG	MDRG A	All diagnosis related groups belonging to Pre-MDC A11: HA, S; A95: S; A97: COPD, S	COPD, HA, S
	MDRG B	All diagnosis related groups belonging to MDC1	S
	MDRG E	All diagnosis related groups belonging to MDC4	COPD
	MDRG F	All diagnosis related groups belonging to MDC5	HA
Elixhauser Groups	elix 1, 2	Congestive heart failure, Cardiac arrhythmias	COPD, HA, S
	elix 3	Valvular disease	HA, S
	elix 4 – 6	Pulmonary circulation disorders, Peripheral vascular disorders, Hypertension	COPD, HA, S
	elix 7 – 9	Paralysis, Other neurological disorders, Chronic pulmonary disease	HA, S
	elix 10	Diabetes (uncomplicated)	COPD, HA, S
	elix 11	Diabetes (complicated)	HA, S
	elix 12, 13	Hypothyroidism, Renal failure	COPD, HA, S
	elix 14	Liver disease	HA, S
	elix 18	Metastatic cancer	COPD, S
	elix 19	Solid tumour without metastasis	COPD, HA, S
	elix 20	Rheumatoid arthritis/collagen vascular diseases	S
	elix 21	Coagulopathy	HA, S
	elix 23 – 24	Weight loss, Fluid and electrolyte disorders	COPD, HA, S
	elix 26	Deficiency anaemia	HA, S
	elix 27, 30	Alcohol abuse, Depression	COPD, HA, S

Note. HA: heart attack data set; S: Stroke data set; MDC: major diagnostic category; MDRG: main diagnosis-related group; MDRG B: B02, B04, B20, B36, B70; MDRG E: E36, E65, E90; MDRG F: F24, F35, F36, F41, F60
