# Supplementary material for: Accounting for clustering in automated variable selection using hospital data: a comparison of different LASSO approaches
Source: BMC Med Res Methodol. 2023 Nov 25;23:280. doi: 10.1186/s12874-023-02081-6 (PMC10675967; doi:10.1186/s12874-023-02081-6)
Supplement: Supplementary file 1 — Additional file 1. [file 12874_2023_2081_MOESM1_ESM.zip › Appendix_Table2.pdf]

Table A.2 Variable importances for the CV selected model concerning stroke and heart attack data for the DV Duration Stay.

Variable	no hosps	hosps fixed	hosps random
**Stroke data**			
Internal transfer	100% (.27)	100% (.25)	100% (.24)
Planned admission	100% (.56)	100% (.22)	0% (–)
elix 23	0% (–)	100% (.12)	100% (.12)
elix 7	0% (–)	100% (.11)	80%(.09)
elix 24	0% (–)	80%(.10)	90%(.10)
elix 6	0% (–)	90% (.10)	50% (.09)
MDRG B04	60% (-.14)	80% (-.10)	0% (–)
Part surgical	50% (.16)	40% (.14)	20% (.11)
Emergency	90% (.17)	0% (–)	0% (–)
MDC 1	10% (-.15)	40% (-.15)	40% (-.14)
MDRG B70	10% (-.17)	60% (-.15)	20% (-.10)
From home	60% (-.14)	0% (–)	0%(–)
From hospital	20% (.13)	0% (–)	0% (–)
elix30	0% (–)	10% (.08)	0% (–)
**Heart attack data**			
MDRG F24	100% (-.45)	100% (-.51)	100% (-.46)
Transfer 24h	100% (-.45)	100% (-.16)	100% (-.10)
elix 1	60% (.11)	100% (.13)	100% (.12)
elix 23	0% (–)	100% (.10)	80% (.09)
Part surgical	60% (.53)	10% (.66)	10% (.45)
Part medical	40% (-.47)	90% (-.56)	20% (-.11)
Emergency	0% (–)	100% (-.13)	0% (–)
Planned admission	100% (.24)	0% (–)	0% (–)
Pre-MDC	0% (–)	100% (.11)	0% (–)
Doctor	0% (–)	10% (.09)	0% (–)

Note. Rates of being among the five most important variables (according to absolute values of regression coefficients) across the 20 sub-samples in percent; in brackets: mean estimated regression coefficient over all sub-samples.
