# Supplementary figures and images for: Accounting for clustering in automated variable selection using hospital data: a comparison of different LASSO approaches
Source: BMC Med Res Methodol. 2023 Nov 25;23:280. doi: 10.1186/s12874-023-02081-6 (PMC10675967; doi:10.1186/s12874-023-02081-6)

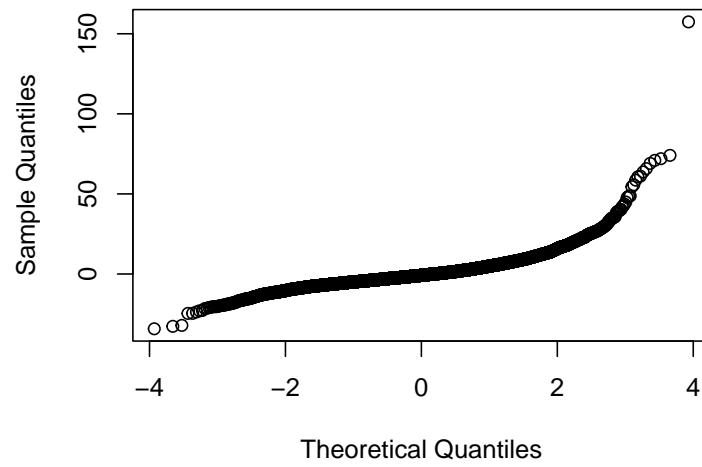

**Figure A.1** QQ-plot of model residuals for raw DV *Duration Stay*

Supplement: Supplementary file 1 — Additional file 1. [file 12874_2023_2081_MOESM1_ESM.zip › Appendix_Figure1.pdf]

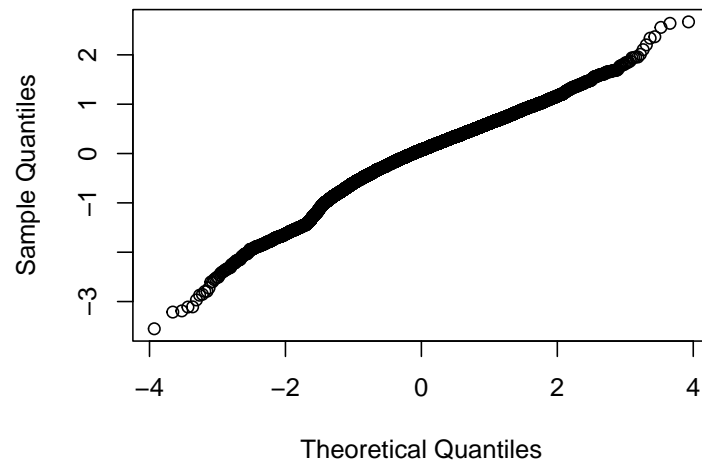

**Figure A.2** QQ-plot of model residuals for log-transformed DV *Duration Stay*

Supplement: Supplementary file 1 — Additional file 1. [file 12874_2023_2081_MOESM1_ESM.zip › Appendix_Figure2.pdf]

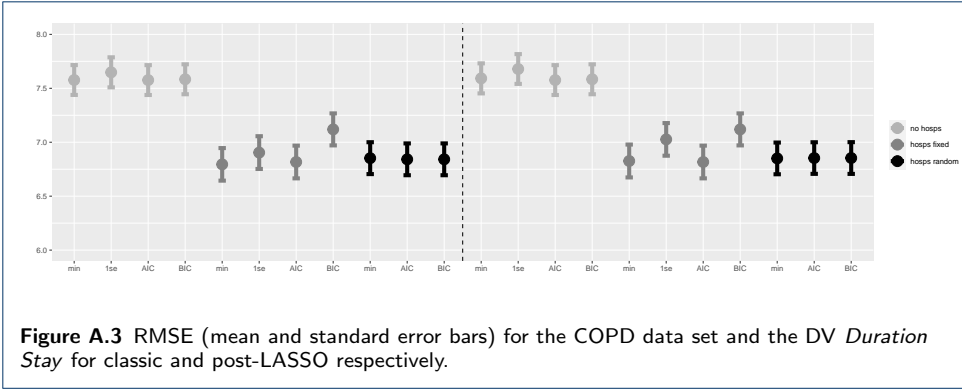

Supplement: Supplementary file 1 — Additional file 1. [file 12874_2023_2081_MOESM1_ESM.zip › Appendix_Figure3.pdf]
